# Neutrophil-and-monocyte-to-lymphocyte ratio is positively associated with elevated prostate-specific antigen levels and high-risk prostate cancer: evidence from the NHANES (2003–2008)

**DOI:** 10.3389/fcell.2025.1573932

**Published:** 2025-04-25

**Authors:** Shang Gao, Ping Jiang, Renli Tian

**Affiliations:** ^1^ Department of Urology, General Hospital of Northern Theater Command, Shenyang, Liaoning, China; ^2^ Department of Graduate School, China Medical University, Shenyang, China

**Keywords:** NMLR, TPSA, HRPCa, NHANES, inflammation

## Abstract

**Introduction:**

Prostate cancer (PCa) is a prevalent malignancy in men globally. The total prostate-specific antigen (TPSA) test is essential for PCa screening. The neutrophil-and-monocyte-to-lymphocyte ratio (NMLR) has emerged as a potential biomarker for various diseases, but its relationship with PCa and TPSA is yet to be studied. This research aims to explore the connection between NMLR and TPSA levels, as well as high-risk prostate cancer (HRPCa), utilizing data from the National Health and Nutrition Examination Survey (NHANES) spanning 2003 to 2008.

**Methods:**

The study included 4,248 U.S. adult males. NMLR was calculated as the ratio of the combined counts of peripheral neutrophils and monocytes to the counts of peripheral lymphocytes. Weighted multiple linear and logistic regression models were used to analyze the relationship between NMLR, TPSA levels, and HRPCa.

**Results:**

A significant positive association was found between elevated NMLR levels and increased TPSA (β = 0.35, 95% CI: 0.21–0.49), as well as higher odds of HRPCa (OR = 2.04, 95% CI: 1.55–2.68). Smooth curve fitting results indicate that there is a nonlinear positive correlation between NMLR and TPSA, as well as between NMLR and HRCa.

**Conclusion:**

This study reveals a significant relationship between NMLR, TPSA levels, and HRPCa odds among U.S. males, suggesting that NMLR could be a valuable biomarker for assessing PCa risk, underscoring inflammation’s role in prostate health. Further research is encouraged to explore its applications in early detection, treatment efficacy evaluation and risk stratification.

## 1 Introduction

Prostate cancer (PCa)ranks among the most prevalent malignancies affecting men globally, with approximately 1.6 million new cases and around 366,000 fatalities reported each year (Fitzmaurice et al., 2017). Additionally, it ranks as the leading malignancy among men in the United States, representing 29% of all cancer diagnoses (Siegel et al., 2024). Screening may reduce the risk of distant metastases (Schröder et al., 2012; Hugosson et al., 2019). The measurement of serum prostate specific antigen (PSA) is extensively employed and considered essential in facilitating the early detection of prostate cancer (Catalona et al., 1994). Patients are at an high risk of PCa (HRPCa) if their total PSA (TPSA) exceeds 10, or if their TPSA falls between 4 and 10 with free PSA (fPSA) constituting less than 25% of the TPSA (Catalona et al., 1994; 1998). Inflammation plays a crucial role in the pathophysiological processes associated with PCa (Klein and Silverman, 2008; Caruso et al., 2009). Neutrophils are recognized as a contributing factor in the etiology of prostate cancer (Zhi-Gang and Han-Dong, 2024). Neutrophils may enhance tumor growth and metastasis by promoting angiogenesis, forming extracellular traps, and utilizing neutrophil elastase (Cools-Lartigue et al., 2013; Gong et al., 2013; S et al., 2015). Monocytes serve as precursor cells for macrophages. Macrophages have the capacity to enhance angiogenesis in tumors (Chen and Bonaldo, 2013). Low levels of CD4 T lymphocytes are associated with tumor recurrence (Hicks et al., 1989; McMillan et al., 1997).

The monocyte-to-lymphocyte ratio (MLR) and neutrophil-to-lymphocyte ratio (NLR) are predictive indicators for PCa(Wang et al., 2024). NMLR, defined as the ratio of the combined counts of peripheral neutrophils and monocytes to peripheral lymphocyte counts (equivalent to the sum of MLR and NLR), is associated with the prognosis of various diseases (Yan et al., 2020; Zhao et al., 2023; Guo et al., 2024; Xia et al., 2024). Nonetheless, no pertinent research has been conducted on the markers NMLR and PSA, nor on the association between NMLR and PCa. This study aims to examine the associations of NMLR with serum PSA and HRPCa, utilizing data from the National Health and Nutrition Examination Survey (NHanes).

NHANES offers a significant resource for exploring this association within a large, nationally representative sample. This investigation utilizes data from NHANES cycles from 2003 to 2008 to analyze this relationship among adult men in the United States, considering potential confounding factors to enhance the generalizability of the findings (NHANES Questionnaires, Datasets, and Related Documentation, n.d.). By analyzing NHANES data, we aim to reveal the potential value of NMLR in PCa risk assessment. This study will not only facilitate the early detection of high-risk patients but also improve the diagnostic accuracy and treatment outcomes for PCa, thereby reducing the burden of the disease on patients and society. Furthermore, our research will also provide new ideas and directions for the prevention, screening, and management of PCa, promoting further exploration in this field.

## 2 Methods

### 2.1 Population studies and data source

The NHANES is a comprehensive epidemiological study conducted across the United States, systematically gathering and analyzing cross-sectional health data from2003 to 2008. This research aims to provide in-depth, evidence-based insights into population health dynamics, focusing on compiling objective statistical information to address critical public health concerns. A meticulous, multi-stage sampling methodology was employed, which included personal interviews, clinical evaluations, and laboratory tests. Prior to commencing the study, ethical approval was obtained from the Institutional Review Board (IRB) at the National Center for Health Statistics, and informed consent was secured from all participants. Further information can be found at https://www.cdc.gov/nchs/nhanes/index.htm.

**FIGURE 1:**
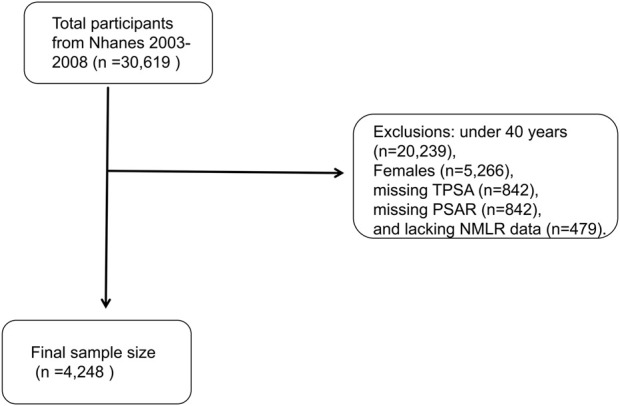
Study process diagram.

During the three assessment cycles, 30,619 individuals were initially recruited for participation. Systematic exclusions were subsequently applied, resulting in the removal of individuals for reasons such as: under 40 years (n = 20,239), Females (n = 5,266), missing TPSA (n = 842), missing PSAR (n = 842) and lacking NMLR data (n = 479). Ultimately, 4,248 individuals were eligible for analysis after these exclusion criteria were implemented (Figure 1).

### 2.2 Exposure variable

Utilizing automated hematology analysis instruments (Beckman Coulter® MAXM instrument) enabled the assessment of monocyte, lymphocyte, and neutrophil counts in a blood count (https://wwwn.cdc.gov/Nchs/Data/Nhanes/Public/2001/DataFiles/L25_B.htm). The NMLR was calculated by taking the ratio of the summed counts of peripheral neutrophils and monocytes to the counts of peripheral lymphocytes.

### 2.3 Outcome variable

Given that the identification of PCa populations in NHANES is based on medical history, the disease course and associated treatments can influence cross-sectional studies. Therefore, we selected TPSA and HRPCa, determined from literature, as the dependent variables. HRPCa is defined as a TPSA level exceeding 10 or a TPSA level between 4 and 10, where fPSA comprises less than 25% of the TPSA. TPSA concentrations were determined using the Beckman Access Immunoassay System’s Hybritech PSA method, which automatically detects light production in reacted samples. The Access Hybritech test, employing a two-site immuno-enzymatic “sandwich” assay, was utilized for measuring fPSA concentrations. Missing PSA data is attributable to NHANES’s eligibility criteria for PSA measurements. PSA testing was conducted in male participants aged 40 years and above. However, those who reported any of the following were deemed ineligible: current infection or inflammation of the prostate gland, a rectal exam within the past week, a prostate biopsy within the past month, a cystoscopy within the past month, or a history of prostate cancer (https://wwwn.cdc.gov/Nchs/Data/Nhanes/Public/2001/DataFiles/L11PSA_B.htm).

### 2.4 Covariates

Covariates encompass factors such as age, race, educational attainment, marital status, BMI, history of alcohol use, smoking status, diabetes, hypertension, stroke, coronary heart disease, the ratio of family income to poverty (PIR), family history of prostate cancer and various biochemical markers, including blood urea nitrogen (mmol/L), cholesterol (mmol/L), total bilirubin (umol/L), and creatinine (µmol/L). Hypertension is defined as having “ever been told by a doctor or other health professional that you had hypertension,” with responses classified as either yes or no. Diabetes is characterized by the statement “Doctor told you have diabetes,” with responses categorized as yes, no, or borderline. The definitions of stroke and coronary heart disease are derived from similar questions. Smoking is defined as having smoked a minimum of 100 cigarettes in one’s lifetime, while alcohol consumption is defined as having had at least 12 alcoholic drinks within a year. Marital status is divided into two categories: Married/Living with partner and never married/Widowed/Divorced/Separated. Education level is classified into three groups: below high school, high school or equivalent, and above high school. For stratified analyses, specific criteria were applied to continuous variables such as age and PIR. PIR is categorized into three levels: less than 1.5, 1.5 to 3.5, and 3.5 or greater. Age (years) is divided into three brackets: 40 to 64, and 65 or older.

### 2.5 Statistical analysis

In view of the fact that NMLR does not follow a normal distribution, this paper applies a logarithmic transformation (log_e_ (NMLR)) to the NMLR used in the analysis, making it closer to a normal distribution and thereby ensuring the effectiveness and accuracy of subsequent analyses.

Acknowledging the complex sampling design, we utilized the survey R package to perform a weighted population analysis stratified by NMLR tertiles. For continuous data, survey-weighted linear regression was implemented, while a survey-weighted Chi-square test was employed to evaluate differences among the three groups based on NMLR levels. Continuous variables are reported as weighted means with 95% confidence intervals (CIs), and categorical variables are summarized with weighted percentages along with their corresponding 95% CIs. We also applied weighted multiple linear regression to analyze the relationship between NMLR and TPSA, and employed weighted logistic regression to explore the association between NMLR and HRPCa.

Given NHANES’s intricate survey design, appropriate weights were applied throughout our analyses. The study utilized three comprehensive models: Model one was unadjusted; Model 2 controlled for age, race, educational attainment, and marital status; and Model 3 incorporated a wider range of factors including age, race, educational level, PIR, marital status, smoking and alcohol use, BMI, stroke, coronary heart disease, blood urea nitrogen, cholesterol, total bilirubin, creatinine, hypertension, family history of prostate cancer and diabetes status. Adjusting for covariates in Model 3, we examined potential nonlinear relationships by constructing smooth curves using generalized additive models. We further performed saturation threshold effect analyses employing piecewise regression, utilizing the log-likelihood ratio test to compare a one-line (non-segmented) model against the segmented regression model, alongside a two-step recursive method. Additionally, subgroup analyses and interaction assessments were conducted. All incomplete covariate data were imputed through a random forest method. Statistical analyses were performed using EmpowerStats (http://www.empowerstats.com, X&Y Solutions, Inc.) and the R statistical package, with a P-value of less than 0.05 considered statistically significant.

## 3 Results

### 3.1 Participant baseline characteristics

This study comprised 4,248 adult male participants. Compared with the lowest NMLR group, the highest NMLR group had higher HRPCa, age ≥65, Non-Hispanic White, Other Race, diabetes, coronary heart disease, stroke, smoking, hypertension, TPSA, blood urea nitrogen, total bilirubin, and creatinine (p < 0.05). Conversely, compared with the lowest NMLR group, Mexican American, Other Hispanic, Non-Hispanic Black, PIR ≥3.5, married/cohabiting, and cholesterol were lower in the highest group (p < 0.05) (Table 1).

**TABLE 1:** Baseline characteristics.

	Low	Middle	High	P Value
HRPCa				<0.0001
Yes	2.6 (2.0,3.5)	4.3 (3.3,5.5)	7.7 (6.2,9.6)	
No	97.4 (96.5,98.0)	95.7 (94.5,96.7)	92.3 (90.4,93.8)	
PIR				0.0049
<1.5	17.8 (15.6,20.2)	15.8 (13.6,18.2)	18.4 (15.5,21.6)	
1.5–3.5	30.4 (27.4,33.5)	28.3 (24.6,32.3)	34.9 (31.0,39.1)	
≥3.5	51.8 (47.6,56.0)	55.9 (51.6,60.2)	46.7 (41.4,52.1)	
Age				<0.0001
<65	85.2 (83.1,87.1)	79.2 (76.7,81.6)	66.9 (63.7,69.9)	
≥65	14.8 (12.9,16.9)	20.8 (18.4,23.3)	33.1 (30.1,36.3)	
BMI				0.3341
<25	20.9 (17.8,24.5)	20.3 (18.0,23.0)	23.0 (20.1,26.1)	
25–30	46.1 (42.1,50.2)	42.9 (39.8,46.1)	41.6 (37.1,46.2)	
≥30	32.9 (29.9,36.1)	36.7 (33.1,40.5)	35.4 (31.2,39.9)	
Race				<0.0001
Mexican American	6.5 (4.7,8.9)	6.5 (4.9,8.6)	4.3 (3.1,5.9)	
Other Hispanic	4.0 (2.6,6.0)	3.3 (2.2,5.0)	1.5 (0.9,2.5)	
Non-Hispanic White	68.0 (62.9,72.6)	79.4 (75.4,82.8)	83.8 (80.0,86.9)	
Non-Hispanic Black	16.7 (13.7,20.1)	6.2 (4.8,8.0)	4.9 (3.7,6.4)	
Other Race - Including Multi-Racial	4.8 (3.2,7.3)	4.6 (3.1,6.7)	5.5 (3.8,7.8)	
Marital status				0.0119
Married/Living with partner	78.3 (75.7,80.8)	78.6 (75.9,81.1)	73.6 (70.2,76.8)	
Never married/Widowed/Divorced /Separated	21.7 (19.2,24.3)	21.4 (18.9,24.1)	26.4 (23.2,29.8)	
Level of education (%)				0.4005
Below high school	20.4 (17.7,23.5)	16.7 (14.6,19.2)	18.8 (15.8,22.1)	
High school or equivalent	24.9 (21.6,28.4)	26.7 (23.6,30.1)	26.0 (23.2,29.0)	
Above high school	54.7 (50.4,58.9)	56.5 (52.6,60.4)	55.2 (50.9,59.5)	
Alcohol				0.9406
At least 12 alcohol drinks/1 year	83.0 (79.5,86.0)	83.1 (80.0,85.7)	83.6 (79.6,87.0)	
Less than12 alcohol drinks/1 year	17.0 (14.0,20.5)	16.9 (14.3,20.0)	16.4 (13.0,20.4)	
Diabetes status				0.0059
Yes	10.1 (8.1,12.5)	10.4 (8.8,12.4)	14.0 (12.5,15.7)	
No	88.1 (85.5,90.3)	88.1 (86.0,89.9)	83.2 (81.2,85.0)	
Borderline	1.8 (1.1,2.9)	1.4 (0.9,2.4)	2.8 (1.8,4.4)	
Coronary heart disease				<0.0001
Yes	5.0 (3.7,6.7)	6.4 (5.3,7.8)	12.0 (10.1,14.2)	
No	95.0 (93.3,96.3)	93.6 (92.2,94.7)	88.0 (85.8,89.9)	
Stroke				0.0005
Yes	2.6 (1.8,3.9)	3.1 (2.4,4.0)	5.3 (4.2,6.6)	
No	97.4 (96.1,98.2)	96.9 (96.0,97.6)	94.7 (93.4,95.8)	
Smoking status (%)				0.0039
at least 100 cigarettes in life	61.6 (57.5,65.5)	55.1 (50.8,59.3)	64.7 (60.3,68.8)	
Less than 100 cigarettes in life	38.4 (34.5,42.5)	44.9 (40.7,49.2)	35.3 (31.2,39.7)	
High blood pressure				<0.0001
Yes	35.1 (31.1,39.4)	39.0 (35.9,42.3)	46.3 (43.1,49.6)	
No	64.9 (60.6,68.9)	61.0 (57.7,64.1)	53.7 (50.4,56.9)	
Family history of prostate cancer				0.3559
Yes	12.1 (9.7,14.9)	13.9 (12.0,15.9)	14.0 (12.1,16.2)	
No	87.9 (85.1,90.3)	86.1 (84.1,88.0)	86.0 (83.8,87.9)	
TPSA (ng/mL)	1.2 (1.1,1.3)	1.5 (1.4,1.6)	1.8 (1.6,2.0)	<0.0001
Blood urea nitrogen (mmol/L)	4.8 (4.7,4.9)	5.1 (5.0,5.2)	5.5 (5.3,5.6)	<0.0001
Cholesterol (mmol/L)	5.3 (5.2,5.4)	5.3 (5.2,5.3)	5.1 (5.0,5.2)	0.0013
Total bilirubin (umol/L)	14.3 (13.9,14.6)	14.7 (14.3,15.0)	14.8 (14.5,15.2)	0.0449
Creatinine (µmol/L)	89.1 (87.7,90.6)	91.1 (89.6,92.7)	94.4 (92.5,96.2)	0.0003

HRPCa, High-Risk Prostate Cancer; BMI, body mass index; PIR, the ratio of family income to poverty; TPSA, total Prostate Specific Antigen.

Data in the table.

For continuous variables: survey-weighted mean (95% CI), P-value was by survey-weighted linear regression (svyglm).

For categorical variables: survey-weighted percentage (95% CI), P-value was by survey-weighted Chi-square test (svytable).

### 3.2 Association of NMLR and both TPSA and HRPCa

We conducted an analysis to explore the association between NMLR levels and TPSA, as detailed in Table 2. In Model 3, after adjusting for a range of covariates—including age, race, level of education, PIR, marital status, smoking status, alcohol status, stroke, BMI, coronary heart disease, blood urea nitrogen, cholesterol, total bilirubin, creatinine, hypertension, family history of prostate cancer and diabetes status—our findings revealed that the significant positive association between NMLR levels and TPSA remained robust (β = 0.35, 95% CI: 0.21–0.49). Notably, when NMLR was categorized into tertiles, individuals in the high tertile demonstrated a markedly high level of TPSA compared to those in the low reference group (β = 0.41, 95% CI: 0.25–0.56). Furthermore, trend analysis indicated a significant escalation in the value of TPSA aligned with rising NMLR levels (P for trend = 0.0001).

**TABLE 2:** Association of NMLR and both TPSA and HRPCa.

Outcome	Exposure	Model 1 β (95% CI)	Model 2 β (95% CI)	Model 3 β (95% CI)
TPSA	NMLR	0.56 (0.39, 0.73)	0.36 (0.20, 0.51)	0.35 (0.21, 0.49)
	Low	Ref.	Ref.	Ref.
	Middle	0.28 (0.15, 0.41)	0.25 (0.12, 0.37)	0.23 (0.10, 0.35)
	High	0.61 (0.43, 0.79)	0.40 (0.25, 0.55)	0.41 (0.25, 0.56)
	P for trend	<0.0001	<0.0001	0.0001
		Model 1 OR (95% CI)	Model 2 OR (95% CI)	Model 3 OR (95% CI)
HRPCa	NMLR	2.67 (2.02, 3.52)	1.92 (1.50, 2.47)	2.04 (1.55, 2.68)
	Low	Ref.	Ref.	Ref.
	Middle	1.64 (1.08, 2.48)	1.57 (1.02, 2.42)	1.52 (0.99, 2.32)
	High	3.09 (2.17, 4.40)	2.43 (1.69, 3.50)	2.56 (1.75, 3.73)
	P for trend	<0.0001	<0.0001	0.0001

HRPCa, High-Risk Prostate Cancer; BMI, body mass index; PIR, the ratio of family income to poverty; TPSA, total prostate specific antigen; OR, odds ratio; CI, confidence interval.

Model 1: no covariates were adjusted.

Model 2: age, race, level of education and Marital status were adjusted.

Model 3: age, race, level of education, PIR, marital status, smoking status, alcohol status, stroke; BMI, coronary heart disease, blood urea nitrogen, cholesterol, total bilirubin, creatinine, hypertension, family history of prostate cancer and diabetes status were adjusted.

Similarly, upon adjustment for multiple covariates, our analysis identified a significant positive association between NMLR and HRPCa (OR = 2.04, 95% CI: 1.55–2.68). The tertile analysis, utilizing the low tertile as a reference, revealed a significant increment in the odds of HRPCa in high tertiles (OR = 2.56, 95% CI: 1.75–3.73). Additionally, trend analysis yielded a statistically significant increase in the odds of HRPCa corresponding to higher NMLR levels (P for trend = 0.0001). For further details, refer to Table 2.

### 3.3 Subgroup analyses

Subgroup analyses were conducted to investigate the relationships between NMLR, TPSA, and HRPCa, taking into account various factors such as age, ethnicity, BMI, diabetes status, and marital status, in combination with Model 3. No subgroup was identified to significantly impact the associations between NMLR and TPSA or HRPCa (P > 0.05) (Table 3).

**TABLE 3:** Subgroup analysis.

Subgroup	TPSA		HRPCa	
	Adjust model β (95% CI)	P For interaction	Adjust model OR (95% CI)	P For interaction
Stratified by age		0.1744		0.277
<65	0.28 (0.15, 0.41)		2.53 (1.51, 4.23)	
≥65	0.50 (0.20, 0.80)		1.80 (1.30, 2.47)	
Stratified by race		0.1922		0.4802
Mexican American	0.31 (−0.06, 0.68)		1.54 (0.64, 3.72)	
Other Hispanic	1.00 (0.41, 1.58)		5.34 (0.79, 35.90)	
Non-Hispanic White	0.31 (0.12, 0.49)		2.04 (1.42, 2.95)	
Non-Hispanic Black	0.36 (−0.22, 0.94)		1.63 (0.91, 2.92)	
Other Race - Including Multi-Racial	0.80 (−0.12, 1.71)		6.07 (1.02, 36.12)	
Stratified by BMI		0.7248		0.5411
<25	0.29 (0.03, 0.55)		1.93 (1.32, 2.80)	
25–30	0.44 (0.14, 0.73)		1.86 (1.15, 3.00)	
≥30	0.28 (0.06, 0.49)		2.74 (1.54, 4.85)	
Stratified by diabetes		0.5507		0.4023
Yes	0.65 (0.04, 1.26)		1.20 (0.51, 2.81)	
No	0.31 (0.17, 0.45)		2.19 (1.61, 2.98)	
Borderline	0.29 (−0.38, 0.96)		2.53 (0.85, 7.57)	
Stratified by marital status		0.7882		0.6693
Married/Living with partner	0.36 (0.20, 0.52)		2.14 (1.51, 3.03)	
Never married/Widowed/Divorced /Separated	0.32 (0.04, 0.60)		1.80 (0.95, 3.41)	

HRPCa, High-Risk Prostate Cancer; BMI, body mass index; PIR, the ratio of family income to poverty; TPSA, total prostate specific antigen; OR, odds ratio; CI, confidence interval.

For the age subgroup, race, level of education, PIR, marital status, smoking status, alcohol status, stroke; BMI, coronary heart disease, blood urea nitrogen, family history of prostate cancer, cholesterol, total bilirubin, creatinine, hypertension and diabetes status were adjusted.

For the race subgroup, age, level of education, PIR, marital status, family history of prostate cancer, smoking status, alcohol status, stroke, BMI, coronary heart disease, blood urea nitrogen, cholesterol, total bilirubin, creatinine, hypertension and diabetes status were adjusted.

For the BMI, subgroup, age, race, level of education; PIR, marital status, smoking status, family history of prostate cancer, alcohol status, stroke, coronary heart disease, blood urea nitrogen, cholesterol, total bilirubin, creatinine, hypertension and diabetes status were adjusted.

For the diabetes status subgroup, age, race, level of education, PIR, marital status, smoking status, alcohol status, stroke; BMI, coronary heart disease, family history of prostate cancer, blood urea nitrogen, cholesterol, total bilirubin, hypertension and creatinine were adjusted.

For the marital status subgroup, age, race, level of education, PIR, smoking status, alcohol status, stroke; BMI, coronary heart disease, blood urea nitrogen, cholesterol, family history of prostate cancer, total bilirubin, creatinine, hypertension and diabetes status were adjusted.

### 3.4 Nonlinear relationships

**FIGURE 2:**
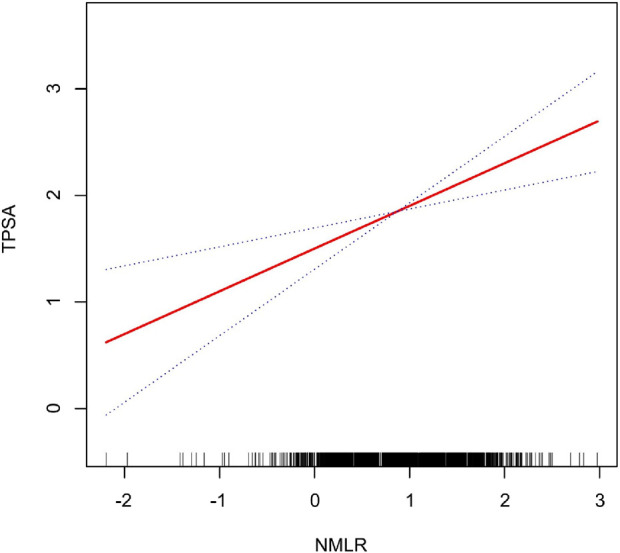
The NMLR and the TPSA exhibit a non-linear association. This relationship is visualized by the solid red curve, which represents the smooth fitting of the variables. The blue dashed lines delineate the 95% confidence interval for the fitted curve.

**FIGURE 3:**
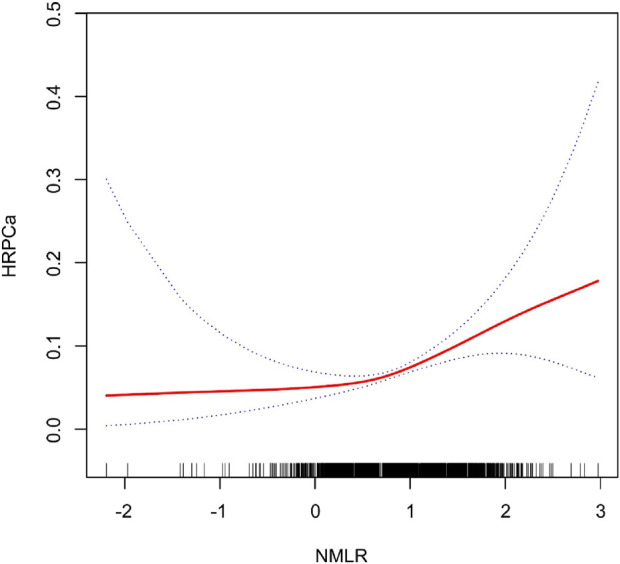
The NMLR and the HRPCa exhibit a non-linear association. This relationship is visualized by the solid red curve, which represents the smooth fitting of the variables. The blue dashed lines delineate the 95% confidence interval for the fitted curve.

Figures 2, 3 illustrate the non-linear positive relationship between NMLR dosage and TPSA levels, as well as the odds of HRPCa, based on analyses using generalized additive models.

## 4 Discussion

In this study involving 4,248 adult male participants, we investigated the relationship between NMLR and both TPSA levels and HRPCa. Our analysis demonstrated a robust positive association between elevated NMLR and increased TPSA, after comprehensive adjustments for various confounders. Trend analysis revealed a significant increase in TPSA correlated with rising NMLR levels (P for trend = 0.0001). Additionally, the analysis indicated that higher NMLR levels are associated with an increased odds of HRPCa (OR = 2.04, 95% CI: 1.55–2.68), particularly in individuals within the highest tertiles (OR = 2.56, 95% CI: 1.75–3.73), reinforcing the strength of this association. Subgroup analyses further revealed that no subgroup was identified to significantly influence the relationship between NMLR and HRPCa risk. Non-linear associations indicate a positive correlation between NMLR dosage and TPSA levels, as well as the odds of HRPCa.

NMLR, as an index derived from complete blood counts, has high accessibility and broad application potential. It has been linked to a range of diseases, including psoriasis, erectile dysfunction, and sarcopenia (Zhao et al., 2023; Guo et al., 2024; Zhang et al., 2024). This makes NMLR a promising biomarker in clinical practice. PSA currently plays an indispensable role in the screening of PCa. However, current research on the relationship between NMLR, PSA, and HRPCa is still relatively limited, highlighting the need for more in-depth exploration in this field. Our research contributes to this gap by demonstrating a positive correlation between elevated NMLR levels and TPSA as well as HRPCa. This finding highlights the potential of NMLR to serve as an additional biomarker, providing new insights into early detection strategies for PCa, treatment efficacy evaluation, risk stratification and potentially guiding clinical decision-making. Current literature provides evidence that inflammation plays a key role in the pathogenesis of PCa (Zarif et al., 2019; Maynard et al., 2022; Novysedlak et al., 2024). Our research findings are consistent with this, indicating that the inflammatory status reflected by NMLR may be an important factor influencing the development of PCa. Moreover, certain blood cell-derived indicators, such as the Systemic Immune-Inflammation Index, NLR, and MLR, demonstrate a positive correlation with PCa and PSA levels (Xu et al., 2021; Tang et al., 2024; Wang et al., 2024). Therefore, as the sum of NLR and MLR, NMLR may hold significant clinical value in identifying early PCa, assessing the inflammatory status of PCa patients, and further stratifying their risk. The role of monocytes and macrophages in tumor pathology emphasizes the importance of immune response in cancer progression. Monocytes differentiate into macrophages and produce migration inhibitory factor (MIF), which functionally inactivates the tumor suppressor factor p53, thereby obstructing DNA damage repair mechanisms and leading to the accumulation of mutations (Calandra and Bucala, 1997; Hudson et al., 1999). Furthermore, macrophages generate elevated levels of reactive oxygen and nitrogen species that can form peroxynitrite, leading to mutagenic events in epithelial and adjacent cells through DNA interaction (Weitberg et al., 1983; Maeda and Akaike, 1998; Pollard, 2004). Macrophages also play an essential role in tumor angiogenesis, primarily through the production of Vascular Endothelial Growth Factor (VEGF) (Leek et al., 2000; Lewis et al., 2000). Additionally, they secrete matrix metalloproteinases (MMPs), contributing to tumor progression and metastasis (Egeblad and Werb, 2002). Similarly, neutrophils share analogous mechanisms in tumor pathophysiology; their release of reactive oxygen species, notably hypochlorous acid, poses risks of DNA damage, posing a significant source of genotoxicity within the tumor microenvironment (Güngör et al., 2010). Neutrophils further promote tumor growth and angiogenesis through the secretion of factors like MMP-9 and neutrophil elastase (Gong et al., 2013; Deryugina et al., 2014). Low levels of CD4 T lymphocytes are linked to an increased risk of tumor recurrence (Hicks et al., 1989; McMillan et al., 1997). Cytotoxic T lymphocytes have shown efficacy in treating metastatic PCa with bone metastases by reducing bone metastasis and significantly lowering serum PSA levels (Shi et al., 2018).

The primary strengths of our study include its large, nationally representative sample, which enhances the generalizability of our findings to the broader U.S. male population. We employed weighted linear regression and weighted logistic regression models that accounted for multiple confounders, complemented by nonlinear modeling, all of which bolster the credibility of our results.

Nonetheless, several limitations should be noted. First, the cross-sectional nature of our analysis restricts our ability to infer a causal relationship; future research employing longitudinal or causal designs (e.g., Mendelian randomization) is necessary, particularly for establishing a corresponding GWAS database for NMLR. Additionally, we cannot entirely rule out the possibility of residual confounding by unmeasured variables. Moreover, due to the NHANES inclusion criteria, missing PSA data may introduce selection bias into the study. In particular, participants excluded from PSA testing—those with current prostate inflammation, recent prostate-related procedures, or a history of prostate cancer—often exhibit higher PSA levels and potentially more elevated inflammatory indicators (NMLR). Excluding these individuals effectively removes a subgroup that could strengthen the observed positive association. As a result, this exclusion likely biases the findings toward the null, suggesting that the positive associations observed between NMLR, PSA, and HRPCa may be underestimated. We only used HRPCa as a binary dependent variable, and future analyses should also consider diagnosed PCa for a more comprehensive understanding.

## 5 Conclusion

Our study demonstrates a significant positive correlation between the NMLR and both TPSA levels and the risk of HRPCa among U.S. males. These findings suggest that NMLR may serve as a valuable biomarker for identifying individuals at high risk for prostate cancer. This research emphasizes the importance of inflammation in prostate health and encourages further investigation into the potential role of NMLR in early detection, risk stratification, and treatment efficacy evaluation, especially when it can be combined with traditional screening methods such as PSA.

## Data Availability

The datasets presented in this article are not readily available because In our research, we examined datasets that are publicly accessible. The data can be accessed through the NHANES database: (https://www.cdc.gov/nchs/nhanes/index.htm). Requests to access the datasets should be directed to The data can be accessed through the NHANES database: (https://www.cdc.gov/nchs/nhanes/index.htm).
